# Early identification of treatment non-response in first-episode psychosis

**DOI:** 10.1192/j.eurpsy.2023.15

**Published:** 2023-03-14

**Authors:** Kristin Fjelnseth Wold, Akiah Ottesen, Bärthel Flaaten Camilla, Erik Johnsen, Trine Vik Lagerberg, Kristin Lie Romm, Carmen Simonsen, Torill Ueland, Line Widing, Gina Åsbø, Ingrid Melle

**Affiliations:** 1 NORMENT, Centre of Excellence, Division of Mental Health and Addiction, Oslo University Hospital and Institute of Clinical Medicine, University of Oslo, Oslo, Norway; 2 Norwegian Centre for Violence and Traumatic Stress Studies, Oslo, Norway; 3 Department of Psychology, Faculty of Social Sciences, University of Oslo, Oslo, Norway; 4 NORMENT, Centre of Excellence, Haukeland University Hospital, Bergen, Norway; 5 Department of Clinical Medicine, University of Bergen, Haukeland University Hospital, Bergen, Norway; 6 Division of Psychiatry, Haukeland University Hospital, Bergen, Norway; 7 Early Intervention in Psychosis Advisory Unit for Southeast Norway, Division of Mental Health and Addiction, Oslo University Hospital, Oslo, Norway

**Keywords:** first episode psychosis, recovery, remission, treatment resistance, treatment response

## Abstract

**Background:**

Approximately one-third of patients with psychotic disorders does not respond to standard antipsychotic treatments. Consensus criteria for treatment resistance (TR) may aid the identification of non-response and subsequent tailoring of treatments. Since consensus criteria require stability of clinical status, they are challenging to apply in first-episode psychosis (FEP). This study aims to investigate (a) if an adaptation of consensus criteria can be used to identify FEP patients with early signs of TR (no early clinical recovery—no-ECR) after 1 year in treatment and (b) to what extent differences in antipsychotic treatments differentiate between outcome groups.

**Methods:**

Participants with FEP DSM-IV schizophrenia spectrum disorders were recruited during their first treatment. A total of 207 participated in the 1-year follow-up. Remission and recovery definitions were based on adaptations of the “Remission in Schizophrenia Working Group” criteria and TR on adaptations of the “Treatment Response and Resistance in Psychosis” (TRRIP) working group criteria.

**Results:**

97 participants (47%) could be classified as no-ECR, 61 (30%) as ECR, and 49 (23%) as with partial ECR (P-ECR). Statistically significant baseline predictors of no-ECR matched previously identified predictors of long-term TR. Only 35 no-ECR participants had two adequate treatment trials and met the full TRRIP criteria. 21 no-ECR participants were using the same medication over the follow-up year despite the lack of significant effects.

**Conclusion:**

The difference in the percentage of FEP participants classified as no-ECR versus TR indicates that we may underestimate the prevalence of early TR when using consensus criteria.

## Introduction

Psychotic disorders have a heterogeneous outcome [1], with individual differences in treatment response [2]. As many as 80% of first-episode psychosis (FEP) patients experience complete remission of symptoms. Still, more than 50% experience intermittent long-term psychiatric symptoms, and 20–30% develop stable poor outcomes despite adequate treatment [3]. The outcome trajectories of individual patients are difficult to predict. Accumulating evidence, however, indicates that clinical development over the first years of treatment is critical for longer-term outcomes [4–6]. Identifying early indicators of treatment response is thus essential to avoid prolonged exposure to ineffective treatments.

Until recently, outcome studies in FEP have been hampered by the lack of clear consensus definitions of treatment response and treatment resistance (TR) [7, 8]. This lack has reduced the consistency and reproducibility of study results. The Remission in Schizophrenia Working Group (RSWG) and the Treatment Response and Resistance in Psychosis (TRRIP) working group was established to standardize definitions [7, 8] (Table 1). The RSWG criteria define remission as a state characterized by simultaneous scores of mild or less (e.g., Positive and Negative Syndrome Scale [PANSS] item score ≤ 3) for items mapping positive, negative, and disorganized symptom domains comprised by the diagnostic criteria for schizophrenia (e.g., PANSS items P1, delusions; P2, conceptual disorganization; P3, hallucinatory behavior; N1, blunted affect; N4, passive social withdrawal; N6, lack of spontaneity; and G9, unusual though content). This level of low severity should be maintained for a minimum of 6 months [7, 9]. A review of studies using RSWG criteria reported that remission rates still varied extensively (17–78%), due to differences in sample compositions and follow-up lengths [10].

**Table 1.** RSWG-based criteria for remission and TRRIP criteria for TR.

	RSWG	TRRIP
Symptoms	PANSS 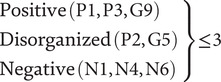	PANSS 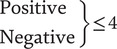
Duration	24 weeks	Minimum 12 weeks
Functioning	Absence of appreciable effects on daily functioning	At least moderate functional impairment
Treatment	No treatment criteria in the definition	At least 2 trials, lasting a minimum of 6 weeks each, with a daily dosage equivalent to 600 mg chlorpromazine, or clozapine

Abbreviations: PANSS, Positive and Negative Syndrome Scale; RSWG, Remission in Schizophrenia Working Group; TR, treatment resistance; TRRIP, Treatment Response and Resistance in Psychosis.

Clinical recovery (CR) is defined as stable symptom remission in the context of regained function over a sustained period of at least 6 months by some- and up to 24 months by other criteria [11, 12, 13]. Recovery rates in FEP vary between 10 and 35%, depending on the definition of recovery and the length of the follow-up period [14, 15]. Long-term CR is predicted by good premorbid functioning, lower levels of psychopathology at baseline, and a shorter duration of untreated psychosis (DUP) [14, 16–23]. Early response to treatment, adherence to medication [14, 24], and signs of early CR have also been found to predict longer-term CR [16, 17].

However, approximately one-third of FEP patients will not benefit from traditional antipsychotic medications and are considered TR [8, 25, 26]. The TRRIP criteria define TR as a sustained lack of remission with persistent functional loss in the context of two adequate trials of different antipsychotic medications [8]. TR is associated with a particularly poor clinical outcome and low quality of life, thus representing a major therapeutic challenge [27–29], and comprising a disproportionate share of the total treatment cost for psychotic disorders [30, 31]. Several studies find that an early lack of treatment response predicts poorer outcomes [32].

Studies show that most TR patients do not respond from the onset of their first treatment, while a smaller group appears to develop TR later on [2, 33, 34]. Although clozapine can be effective for some TR patients [34, 35], there are usually long delays before it is used [1, 35, 36]. Some studies suggest that early initiation of clozapine treatment in patients with early signs of TR may support a better course of illness [2, 33, 37] with the possibility of a critical time window for the clozapine effect [2, 8, 32, 38–40]. Studies imply that such a critical time window in which clozapine is most effective exists [32, 37, 39, 41]; however, the duration of that possible window remains unclear [39]. Based on a reanalysis of a study of clozapine-treated TR patients, Yoshimura et al. [39] suggested a time window of approximately 3 years, analogous to the suggested critical period of 2–5 years for FEP outcomes, based on the observation that 2.8 years delay in initiating clozapine was the most predictive cut-off. Most treatment guidelines thus recommend starting clozapine after two failures of traditional antipsychotic medications. However, studies still show clinicians tend to increase dosages of the standard antipsychotic and/or use polypharmacy instead [33, 34, 42].

Identifying early signs of TR to revise treatment strategies at the earliest possible time could improve treatment outcomes. Still, the early identification of TR is less investigated than early signs of CR. The TRRIP criteria for TR require at least moderate symptoms (e.g., PANSS item scores > 3) and at least moderate functional loss (e.g., GAF/GFS scores ≤ 60) for at least 12 weeks using an antipsychotic in adequate dosage, in the context of at least two previous adequate treatment trials (e.g., more than 6 weeks on an adequate dosage of two different antipsychotics). This implies observation periods of at least 24 weeks on adequate antipsychotic dosages, in addition to the time spent initiating and cross-tapering antipsychotics. It is thus likely that not all non-responding FEP patients will be classified as TR by the TRRIP criteria at a 1-year follow-up. However, the few existing studies of TR in the early phases of FEP have used the standard TRRIP criteria [43].

### Aims

The aims of this study were thus to examine if adapting the consensus criteria, in the form of shortening the required observation periods, could be used to identify early signs of TR in FEP after 1 year of treatment. To answer this research question, we examined: (a) The prevalence of three different early outcomes in a naturalistic FEP sample followed-up after 1 year in treatment. Participants with early signs of clinical recovery (ECR) indicating good treatment response, participants without early signs of clinical recovery (no-ECR) indicating a lack of treatment response, and participants with in-between outcomes, i.e., in partial early clinical recovery (P-ECR). (b) To what extent between-group differences in baseline clinical- and demographic characteristics corresponded to known predictors of longer-term outcomes, thus supporting the validity of the early classification. In addition, we also examined and report on (c) the antipsychotic treatment received over the first year in treatment and (d) the prevalence of patients meeting the full TRRIP criteria for TR, including adequate treatment attempts, at this time point.

## Methods

### Setting and participants

The current study is part of the ongoing Thematically Organized Psychosis (TOP) research study at the Norwegian Centre for Mental Disorders Research (NORMENT). It has a prospective longitudinal observational design using data from baseline and 1-year follow-up.

Participants within the 18- to 65-year age range were recruited from inpatient and outpatient psychiatric units at the major hospitals in the Oslo area between 2002 and 2019. The hospitals cover a catchment area of 660,000 inhabitants and about 88% of the total population of Oslo. The participants met the DSM-IV criteria for schizophrenia, schizophreniform disorder, schizoaffective disorder, and psychotic disorder NOS at baseline and were recruited within their first year of treatment. We included individuals with broad schizophrenia spectrum disorders since the study focuses on the first year of treatment, where diagnosis still can be unstable [44]. Exclusion criteria were the presence of pronounced cognitive deficit (estimated IQ below 70), severe brain injury, or not speaking a Scandinavian language. Due to the high prevalence of illicit substance use in FEP, we have included patients both with and without the use of illicit substances and examined the effect of illicit substance use on outcomes.

A total of 387 participants completed baseline clinical assessments and were eligible for participation in follow-up assessments. Of these, 207 participants completed assessments at 1-year follow-up. We found no significant differences in gender, baseline diagnosis, DUP, positive or negative symptoms at baseline, or premorbid adjustment between completers and non-completers (See Supplementary Figure S1 and Supplementary Table S2 for an overview of attrition).

The study was conducted following the Helsinki declaration and approved by the Regional Committee for Medical and Health Research Ethics in Norway and the Norwegian Data Inspectorate. The participants provided their written informed consent to participate in the study.

### Clinical assessments

Structured clinical assessments were carried out by trained clinical psychologists and medical doctors or psychiatrists. After giving informed consent, participants underwent comprehensive clinical assessments, which took place over several meetings. Diagnoses were established using the Structured Clinical Interview for DSM-IV Axis I disorders (SCID-I), modules A-E (SCID I) [45]. Clinical assessors were trained according to a program developed at UCLA [46]. The inter-rater reliability from the program has previously been found satisfactory, the TOP study had an overall kappa score varying between 0.92 and 0.99 across different assessment teams [47]. A full illness history with information about treatment history was also gathered through interviews, medical journals, blood samples, and adherence questionnaires, in addition to information about education, occupation, and marital status.

Symptoms at baseline and 1-year follow-up were measured based on the Structured Clinical Interview for the Positive and Negative Syndrome Scale (SCI-PANSS) [48], grouped according to the Wallwork five-factor model consisting of a subset of items constituting positive, negative, disorganized, excited, and depressive symptoms [49]. This model is more appropriate than the original three-factor model for assessing FEP populations [49–51].

Premorbid social and academic functioning was measured with the Premorbid Adjustment Scale (PAS) [52], a clinician-rated seven-point scale assessing social- and academic performance over different age groups and where the highest scores indicate the poorest adjustment. The premorbid childhood phase is defined as 0–11 of age. We used only the childhood subscales to ensure that PAS scores were not overlapping with any possible prodromal period preceding the first episode.

DUP was established at baseline and measured in weeks from the onset of psychosis until the start of adequate treatment. Psychosis was defined as having a score ≥4 on the PANSS positive items P1, P3, P5, P6, and G9 for more than 1 week [53].

### Functional assessment

All participants were assessed with the Global Functioning Scale (GFS) [54, 55]. The GFS is a text-revised, two-part scale (split for the level of functioning and symptom burden) corresponding to the Global Assessment of Functioning Scale (GAF) [56, 57]. Participants with a GFS score ≥61 were assessed as having regained functioning at the 1-year follow-up [14, 16]. A score of ≥61 implies that the participant lives independently, takes care of basic needs, is employed, and has meaningful social relationships [54]. In 21 cases, the GFS score was missing at the 1-year follow-up. We then used all available information from the assessments and interviews to classify these cases as above or below 61.

### Outcome groups

The RSWG criteria were used to define remission of symptoms, that is, scores equal to or below three on any of the following PANSS items at the time of follow-up: P1 (delusions), P2 (disorganized thought), P3 (hallucinatory behavior), N1 (affective flattening), N4 (passive social withdrawal), N6 (lack of spontaneity), G5 (bizarre posture), or G9 (unusual thought content) [7]. We used a time criterion for 12 weeks to ensure minimum stability and to mirror the criteria for non-remission stated by the TRRIP instead of the RSWG criterion of 24 weeks. ECR was defined as (a) meeting the criteria for remission and (b) regained functioning, defined as a GFS score ≥61. A time criterion for the latter was not applied. No-ECR was defined as (a) not meeting the previously described remission criteria for more than 12 weeks at follow-up and (b) not regained functioning, defined as a GFS score <61.

P-ECR comprised participants not meeting these criteria, that is, either low symptom levels with low functional levels or high symptom levels with high functional levels.

**Figure 1.**
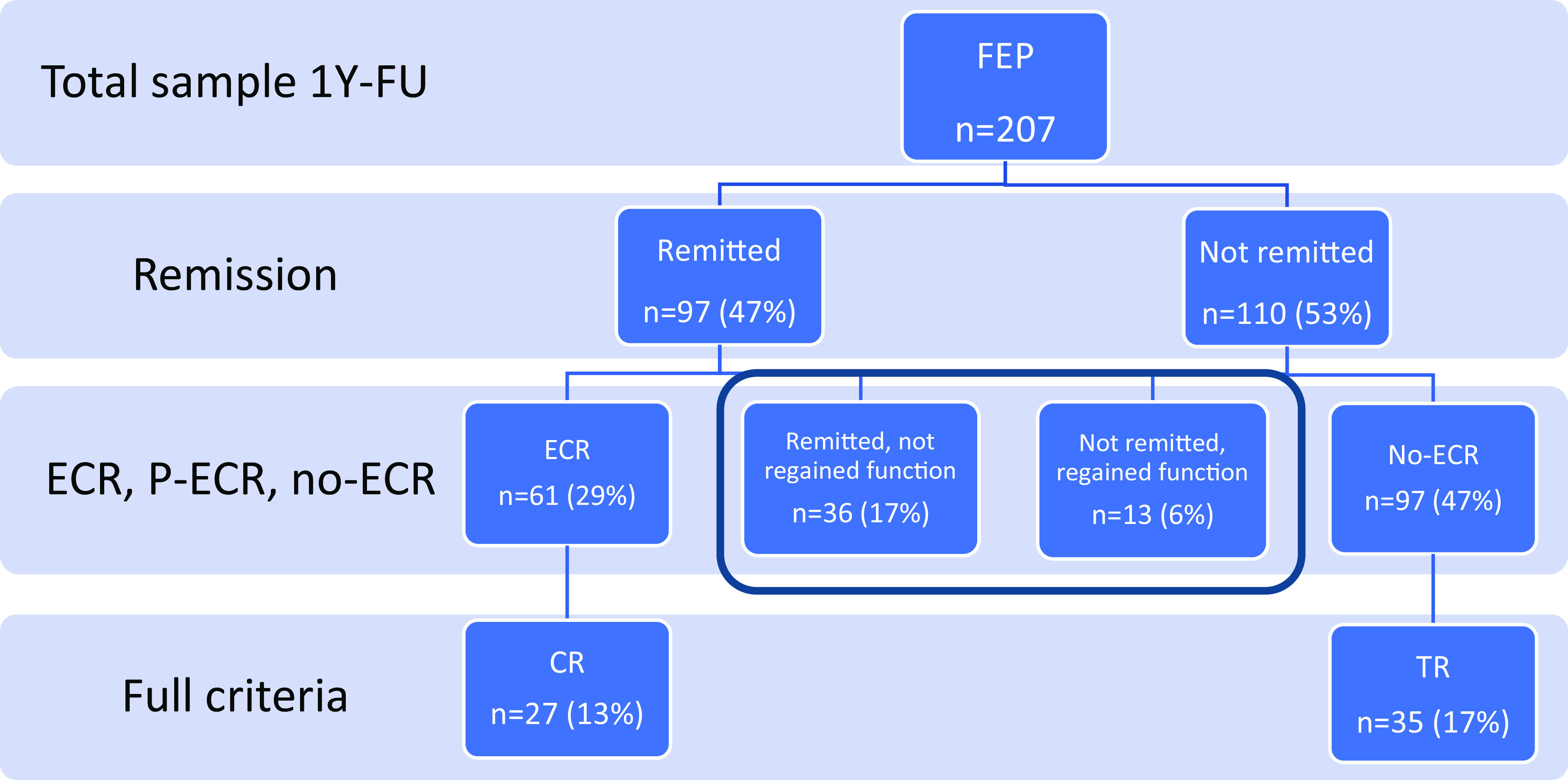
Steps in the definition of the different outcome groups. FEP, first-episode psychosis; CR, clinical recovery; ECR, early clinical recovery; no-ECR, no early clinical recovery; P-ECR, partial early clinical recovery; TR, treatment resistant; 1Y-FU, 1-year follow-up.

We also identified those meeting the TRRIP and RSWG-based CR criteria, including requirements for symptom stability over time and requirements of two adequate treatment trials. Based on the recommendation in the relevant national guideline, we defined an adequate treatment trial as using at least one defined daily dosage (DDD) of antipsychotics [58] for at least 2 weeks or clozapine. The DDD threshold used was based on FEP patients appearing to have a better antipsychotic response than multi-episode patients [59], with the TRRIP definition of adequate dosages higher than those suggested for FEP in treatment guidelines (Figure 1).

### Statistical analyses

We used the TSD (Tjenester for Sensitive Data) facilities at the University of Oslo for safe data storage. For statistical analyses, we used the Statistical Package for the Social Sciences (SPSS) for Windows, IBM SPSS Statistics for Windows, Version 28.0. Armonk, NY: IBM Corp and STATA, version 17.0, StataCorp. 2021. Stata Statistical Software: Release 17. College Station, TX: StataCorp LLC. Data were checked for normality, homogeneity of variance, and outliers.

Group differences in premorbid, sociodemographic, and clinical variables were examined with chi-square tests for categorical variables and ANOVAs with Tukey *post hoc* tests for continuous variables. Kruskal–Wallis tests were performed for nonparametric continuous variables. Group differences in treatment were examined using chi-square tests with Bonferroni corrections.

Ordinal logistic regression analysis, Proportional Odds (PO) model, was performed to investigate predictors of ECR, P-ECR, and no-ECR. The PAS subscales were highly intercorrelated and we chose the scale that differed most between outcome groups (social adjustment). Significant factors that improved model fit were kept for further analyses, using −2 log likelihood, Akaike information criteria (AIC), and Bayesian information criteria (BIC) to assess and compare the model fit. The PO model fit the data better than a multinomial logistic regression model.

We used the margins command in STATA to find predicted probabilities. See Supplementary Table S3 for calculations of the probability of being in each outcome group at the 1-year follow-up for specific scores on the independent variable.

Non-normally distributed variables were log-transformed before inclusion in regression analyses. The predictor variables were tested *a priori* to verify there was no violation of the multicollinearity assumptions. We used a likelihood ratio test and a Brant test to test the proportional odds and parallel regression assumptions. Alfa was set at *p* < 0.05.

## Results

### Outcome groups as defined by adapted criteria

We identified two equally large groups of non-remitted (*n* = 110, 53%) and remitted (*n* = 97, 47%) FEP participants at 1-year follow-up. These could be further subdivided into no-ECR (*n* = 97, 47%), ECR (*n* = 61, 29%), and P-ECR (*n* = 49, 24%). The demographic and clinical characteristics of the outcome groups are presented in Table 2.

**Table 2.** Summary statistics and comparisons of demographic, premorbid, and clinical characteristics of the adapted outcome groups, stratified by outcome group.

	Total sample (*n* = 207)	ECR (*n* = 61, 29%)	P-ECR (*n* = 49, 24%)	No-ECR (*n* = 97, 47%)	ANOVA/Kruskal_Wallis**/chi-square* analyses			*Post hoc*
					*F*(*x* ^2*^)	df	*p*	
Gender (male), *n* (%)	124 (60)	31 (50.8)	25 (51.0)	68 (70.1)	7.91*	2	0.019	ECR, P-ECR < no-ECR
Age, years: mean (SD)	27.2 (7.7)	27.3 (7.9)	25.6 (6.2)	27.9 (8.3)	1.40	2	0.248	n.s.
Age of onset, years: mean (SD)	24.0 (7.4)	25.0 (7.5)	22.9 (7.0)	23.9 (7.4)	1.134	2	0.324	n.s.
Years of education: mean (SD)	13.2 (2.9)	14.4 (2.8)	12.8 (2.7)	12.7 (2.9)	7.326	2	<0.001	ECR > P-ECR, no-ECR
BMI: mean (SD)	24.51 (4.2)	24.33 (4.2)	24.22 (3.6)	24.77 (4.6)	0.339	2	0.713	n.s.
Current relationship, yes (%)	38 (18.4)	15 (24.6)	5 (10.2)	18 (18.6)	3.76*	2	0.153	n.s.
PAS social childhood, mean (SD)	1.16 (1.4)	0.79 (1.0)	0.88 (1.1)	1.55 (1.6)	7.28 W	2	0.001	ECR, P-ECR < no-ECR
PAS academic childhood, mean (SD)	1.60 (1.3)	1.33 (1.0)	1.43 (1.4)	1.86 (1.3)	3.97	2	0.021	ECR < (P-ECR) < no-ECR
Baseline diagnosis (schizophrenia), *n* (%)	107 (51.7)	19 (31.1)	19 (38.8)	69 (71.1)	30.4*	6	<0.001	ECR, P-ECR < no-ECR
DUP (log) median (range)	32 (1300)	8 (1008)	32 (779)	60 (1300)	24.97**	2	<0.001	ECR < P-ECR, no-ECR
GFS: mean (SD)	53.4 (15.0)	70.1 (9.0)	52.4 (12.3)	43.4 (8.8)	167.31 W	2	<0.001	ECR > P-ECR > no-ECR
PANSS positive: mean (SD)	2.48 (1.0)	1.86 (0.8)	2.39 (0.9)	2.90 (1.0)	24.85	2	<0.001	ECR < P-ECR < no-ECR
PANSS negative: mean (SD)	2.06 (0.9)	1.67 (0.7)	2.11 (1.0)	2.29 (0.9)	11.27 W	2	<0.001	ECR < P-ECR, no-ECR
PANSS disorganized: mean (SD)	1.82 (0.8)	1.44 (0.5)	1.79 (0.7)	2.07 (0.9)	16.03 W	2	<0.001	ECR < P-ECR, no-ECR
PANSS depressive: mean (SD)	2.84 (1.0)	2.60 (1.0)	2.63 (1.0)	3.09 (0.9)	6.42	2	0.002	ECR, P-ECR < no-ECR
PANSS excited: mean (SD)	1.39 (0.5)	1.24 (0.4)	1.37 (0.4)	1.49 (0.5)	6.07 W	2	0.003	ECR < (P-ECR) < no-ECR
AUDIT, median (range)	5.0 (33)	5.0 (24)	5.0 (33)	4.0 (33)	1.890**	2	0.389	n.s.
DUDIT, median (range)	0.0 (37)	0.0 (29)	2.5 (37)	0.0 (37)	7.597**	2	0.022	ECR < P-ECR, (no-ECR)

*Note:* */** Continuous variables were compared across groups using one-way ANOVAs with Tukey *post hoc* tests, and categorical variables were compared across groups using chi-squared tests with Bonferroni corrections. For continuous variables, Levene’s test of homogeneity of variances was performed and consequently, Welch test assuming unequal variances was used for variables where the *p*-value of Levenes’ homogeneity test < 0.05. Kruskal–Wallis tests were performed for nonparametric continuous variables.

Abbreviations: AUDIT, Alcohol Use Disorder Identification Test; BMI, body mass index; DUDIT, Drug Use Disorders Identification Test; DUP, duration of untreated psychosis; ECR, early clinical recovery; GFS, Global Functioning Scale; n.s., non-significant; no-ECR, no early clinical recovery; PAS, Premorbid Adjustment Scale; PANSS, Positive and Negative Syndrome Scale; P-ECR, partial early clinical recovery.

There were statistically significant differences between the no-ECR and ECR groups for all investigated demographic, premorbid, and clinical variables, except for age, age of onset, BMI, relationship status, illicit substance use, and alcohol use. The P-ECR group was in-between the ECR and no-ECR groups in all investigated areas.

The ordinal regression analysis (Table 3) indicated that male gender, poorer premorbid social adjustment, DUP, and more positive and negative symptoms at baseline had statistically significant independent contributions to the predicted outcome group membership at 1-year follow-up (−2LL = 341.170, *x* ^2^ = 75.02, df. = 5, *p* < 0.001). A schizophrenia diagnosis did not predict the outcome-group membership after correcting for background variables. The final model containing the complete set of predictors had an 18% improvement in model fit relative to an intercept-only model (McFadden pseudo *R* ^2^ = 0.180).

**Table 3.** Ordinal regression analysis (PO model) showing variables that had statistically significant independent contributions to the predicted outcome group membership at 1-year follow-up.

Parameter	OR	95% CI		Wald *x* ^2^	Significance
Gender (male)	2.17	1.19	3.98	6.310	0.012
PAS social childhood	1.31	1.03	1.65	5.016	0.025
DUP	1.26	1.08	1.48	8.372	0.004
Positive symptoms baseline	2.19	1.57	3.07	20.825	<0.001
Negative symptoms baseline	1.55	1.12	2.15	6.913	0.009

Abbreviations: 95% CI, 95% confidence interval; DUP, duration of untreated psychosis; GFS, Global Functioning Scale; OR, odds ratio; PAS, Premorbid Adjustment Scale.

### Antipsychotic treatment

There were no statistically significant differences between the three outcome groups in the number of adequate treatment trials, the proportion of participants not using any medication at follow-up, or the proportion of participants who had not tried any antipsychotic treatment (Table 4). Approximately one-third of the sample (*n* = 67, 32.4%) was unmedicated at 1-year follow-up, with 10.6% (*n* = 22) reporting no previous use of antipsychotic medication. A significantly larger proportion of the ECR group used the same medication from baseline to follow-up compared to the no-ECR group. Still, 21 participants (22%) of the no-ECR group used the same medication throughout the first year, despite the lack of an adequate clinical response. Five participants used clozapine, four in the no-ECR group and one in the ECR group. There were no differences between the outcome groups regarding which antipsychotic was the first or the second drug of choice (Supplementary Tables S6 and S7 for further details on medication use).

**Table 4.** Summary statistics and comparisons of antipsychotic treatment at 1-year follow-up, stratified by outcome groups.

	Total sample	ECR	P-ECR	No-ECR	Chi-square test*			*Post hoc*
					*x* ^2^	df.	*p*	
Ongoing trial at follow-up *n* (%)	21 (10.1)	1 (1.6)	2 (4.1)	**18 (18.6)**	14.35	2	<0.001	ECR, P-ECR < no-ECR
Used the same medication over first year	67 (32.4)	26 (42.6)	20 (40.8)	**21 (21.6)**	9.62	2	0.008	ECR, P-ECR < no-ECR
No medication at 1-year follow-up	67 (32.4)	24 (39.3)	16 (32.7)	27 (27.8)	2.27	2	0.322	n.s.
No previous use of medication	22 (10.6)	6 (9.8)	4 (8.2)	12 (12.4)	0.66	2	0.718	n.s.
Medication trials *n* (%)								
0	44 (21.3)	10 (16.4)	11 (22.4)	23 (23.7)	6.27	6	0.394	n.s.
1	108 (52.2)	35 (57.4)	29 (59.2)	44 (45.4)	6.27	6	0.394	n.s.
2	50 (24.2)	15 (24.6)	7 (14.3)	28 (28.9)	6.27	6	0.394	n.s.
3	5 (2.4)	1 (1.6)	2 (4.1)	2 (2.1)	6.27	6	0.394	n.s.
Clozapine use *n* (%)	4 (1.9)	1 (1.6)	0 (0.0)	3 (3.1)	1.68	2	0.431	n.s.

Abbreviations: ECR, early clinical recovery; n.s., non-significant; no-ECR, no early clinical recovery; P-ECR, partial early clinical recovery.

* Information about medication was compared across groups using chi-squared tests with Bonferroni corrections.

### Patients meeting the standard outcome criteria

We used information about the duration of the current clinical status and information about medication history, as outlined above, to assess how many would meet the standard RSWG and TRRIP criteria at 1-year follow-up. We found that only 35 (17%) of participants met the full TRRIP criteria for TR and 27 (13%) of participants met the full RSWG-based criteria for CR at this point. The main reason for being in the no-ECR group without meeting the TRRIP criteria for TR was the lack of two adequate trials of antipsychotics (Supplementary Tables S4 and S5 for details). A total of 18 (19%) of the no-ECR group were in an ongoing antipsychotic trial at 1-year follow-up, indicating that this classification might change within the next year.

## Discussion

There is a need to rapidly assess and re-assess treatment effects in the early phases of the treatment for FEP [4, 5, 60]. We here identified two distinct outcome groups showing clear signs of early clinical non-recovery and early clinical recovery, respectively, and a heterogeneous group with partial early clinical recovery. The statistically significant differences in premorbid adjustment, early illness features, and baseline clinical characteristics between the groups are consistent with previously identified predictors of long-term outcome [14, 29, 61] and, more specifically, longer-term CR and TR [10, 14, 16, 18]. This supports the validity of the identified outcome groups as early indicators of long-term outcomes [1, 2, 4, 62]. That a schizophrenia diagnosis *per se* did not have an individual contribution in the regression model shows that the underlying differences are not secondary but integral to the diagnosis.

The predictors also correspond to those identified in a recent FEP study of patients meeting the full TRRIP criteria [43]. In the current study, meeting the full criteria depended, as hypothesized, on having completed two adequate treatment trials before the 1-year follow-up. The prevalence of TR as identified by the TRRIP criteria in the early course of illness should thus be interpreted with some caution, as a large proportion of patients with primary antipsychotic resistance could remain unidentified. This could partly explain why studies report an increase in TR later in the course of illness [2, 63].

Ongoing evaluation of treatment response and a change to clozapine after two adequate but failed treatment trials is part of most treatment guidelines [64, 65]. We thus expected that the participants, although recruited from a naturalistic setting and not a treatment trial, were treated according to the guidelines. However, it appears as if more time was spent on trial and error with different treatment strategies. This could be based on more than just a lack of adherence to guidelines, as shared decision-making is increasingly emphasized. Delays in the initiation of treatment and change of medication could thus be based on the need for psychoeducation and motivation. Previous studies point to non-adherence or discontinuation of antipsychotic medication as essential contributors to relapse and subsequent unfavorable course of illness [66–68]. A slightly surprising finding was the significant proportion of patients not using any antipsychotic medication at 1-year follow-up across all outcome groups, including those with early CR. The high number of unmedicated patients across outcome groups indicates that the outcome differences are not only due to differences in medication use or treatment adherence.

That a high proportion of non-responding first-treatment patients were using the same antipsychotic treatment for a year despite a lack of sufficient response is problematic. It underlines the need for a structured evaluation of treatment response in the early phases to prevent non-responding patients from being exposed to unwanted side effects in the context of little if any, clinical benefit. The proportion using clozapine was also lower than reported in previous studies [33, 69–71]. Fear of severe side effects, clinicians’ unfamiliarity with procedures, and practical inconveniences in addition to the early stage of the disorders are possible reasons for the low number of clozapine trials [33]. However, the good effect of clozapine is illustrated by finding a clozapine user in the ECR group, in line with studies underlining the potential of clozapine use in the early stages of treatment [33, 34, 39].

Psychotic disorders start in young adulthood and many experience relapses and/or continuing symptoms. While the prevalence of psychotic disorders is high, the incidence is however low. Most psychiatrist thus have more experience treating multi-episode patient groups with established partial or complete TR, and this may lead to lower-than-necessary treatment expectations given the potential of good outcomes in well-treated FEP groups. Using predefined criteria for response and non-response adapted to the stage of illness could here provide adequate benchmarks for treatment success.

## Strengths and Limitations

We report on a well-characterized sample of FEP participants in a prospective naturalistic longitudinal design. The participants were recruited without pre-selection from all treatment units in a large catchment area and assessed by trained personnel.

The study protocol and data collection began before the current consensus criteria were published. This could be seen as a limitation because it affects the availability of measurements comprised by the criteria. However, as we still can identify separate outcome groups characterized by well-known predictors of outcome, our findings can also be taken to support the robustness of the adapted criteria.

The retention rate for this study was low, which could affect both statistical power and sample representativity. We found no significant differences between completers and non-completers of the 1-year follow-up for demographics, premorbid adjustment, DUP, and positive and negative symptoms at baseline. The reported retention rate is however higher than in reports of low retention rates in recent longitudinal studies involving patients with psychotic disorders [72]. The low retention rate could also be attributed to the extensive research protocol for the larger translational project that the longitudinal study also was a part of, where participants were asked to join in comprehensive cognitive assessments and MR protocols between the clinical assessments at baseline and 1-year follow-up.

## Conclusions

Using an adapted version of recent consensus criteria for outcome after 1 year of treatment, we identified three outcome groups (no-ECR 47%, ECR 30%, and P-ECR 23%) with statistically significant differences in demographic, premorbid, and clinical characteristics corresponding to predictors of long-term outcome. Using adaptations of consensus criteria thus appears feasible and is reproducible across studies. The resulting groups can be used to more rapidly tailor treatments dependent on clinical status and to study the mechanistic basis of different outcomes at an early stage.

## Data Availability

The anonymized data underlying the current analyses are available on request.

## Supplementary material

For supplementary material accompanying this paper visit http://doi.org/10.1192/j.eurpsy.2023.15. click here to view supplementary material
