# Supplementary material for: Early identification of treatment non-response in first-episode psychosis
Source: Eur Psychiatry. 2023 Mar 14;66(1):e30. doi: 10.1192/j.eurpsy.2023.15 (PMC10134449; doi:10.1192/j.eurpsy.2023.15)
Supplement: Supplementary file 1 [file S0924933823000159sup001.docx]

SUPPLEMETARY MATERIAL

Figure S1: Attrition


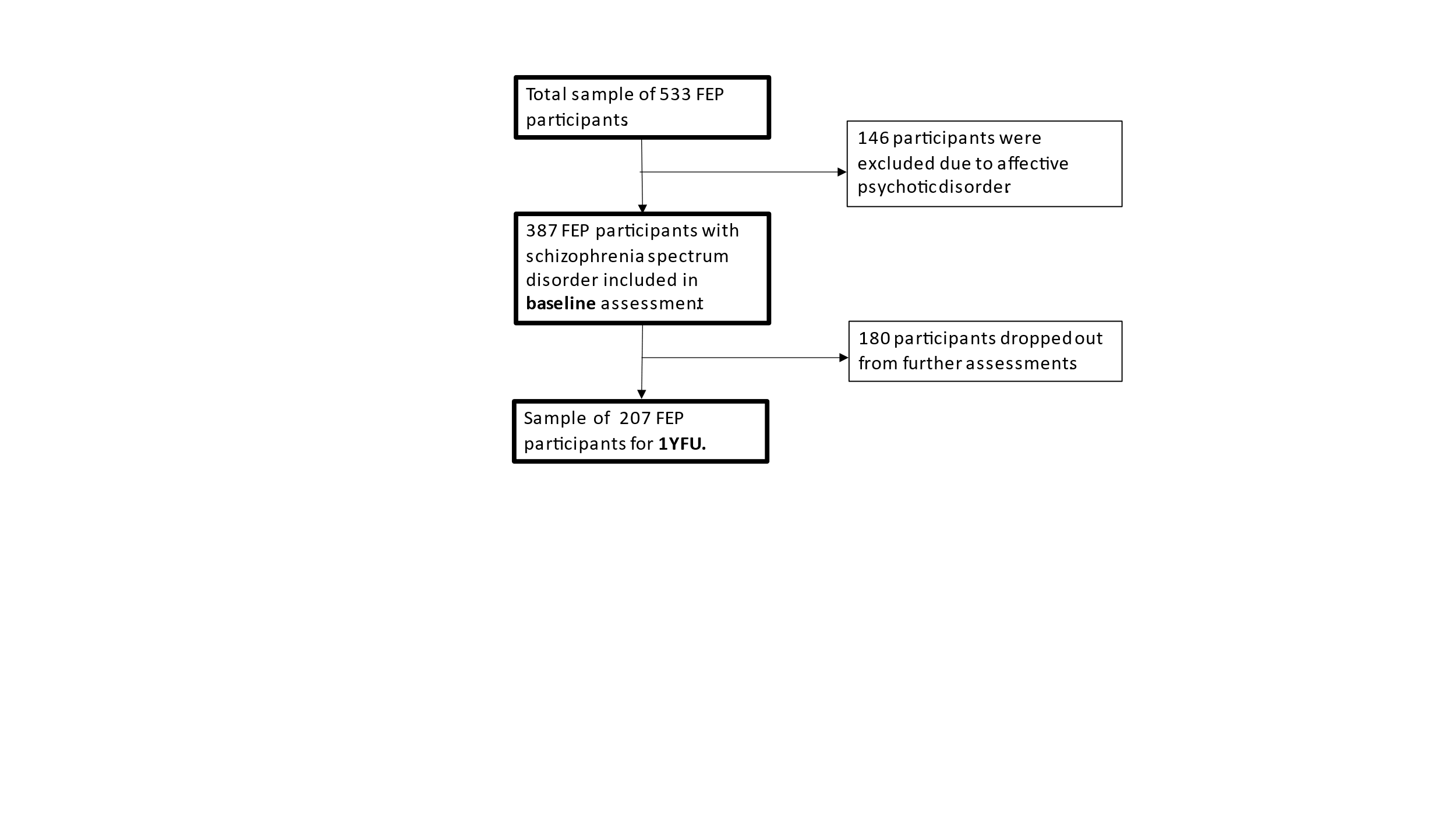


*Abbreviations: FEP, first episode psychosis; 1YFU, one-year follow-up.*

Table S2 Comparisons of baseline data and clinical characteristics between completers and non-completers

|  | Completers | Non-completers | T-test/chi square */Mann Whitney U-test** | | |
| --- | --- | --- | --- | --- | --- |
|  |  |  | T (x^2*^) | df. | P |
| Gender (male), n (%) | 124 (59.9) | 122 (67.8) | 2.58* | 1 | .108 |
| Age, years, mean (SD) | 27.2 (7.7) | 28.8 (8.6) | 1.021 | 385 | .308 |
| PAS social childhood, mean (SD) | 1.16 (1.4) | 1.20 (1.4) | .256 | 369 | .798 |
| PAS academic childhood, mean (SD) | 1.60 (1.3) | 1.83 (1.2) | 1.828 | 366 | .068 |
| Diagnosis (narrow schizophrenia), n (%) | 107 (51.7) | 88 (48.9) | .302* | 1 | .582 |
| DUP, median (range) | 32 (1300) | 44 (1352) | 17451.0** | 1 | .282 |
| GFS baseline, mean (SD) | 46.04 (13.2) | 45.84 (13.0) | -.149 | 385 | .882 |
| PANSS pos baseline, mean (SD) | 2.48 (1.0) | 2.48 (0.9) | .048 | 385 | .962 |
| PANSS neg baseline, mean (SD) | 2.06 (0.9) | 2.22 (1.0) | 1.569 | 385 | .117 |
| PANSS dis baseline, mean (SD) | 1.82 (0.8) | 2.05 (0.9) | 2.764 | 385 | .006 |
| PANSS depr baseline, mean (SD) | 2.84 (1.0) | 2.59 (1.0) | -2.501 | 385 | .013 |
| PANSS excit baseline, mean (SD) | 1.39 (0.5) | 1.49 (0.6) | 1.724 | 385 | .086 |
| Audit score, median (range) | 5 (33) | 5 (36) | 16235.0** | 1 | .454 |
| Dudit score, median (range) | 0 (37) | 0 (40) | 15959.0** | 1 | .926 |

*Abbreviations: PAS, premorbid adjustment scale; DUP, duration of untreated psychosis; GFS, Global Functioning Scale; PANSS, Positive and Negative Syndrome Scale; AUDIT, Alcohol Use Disorder Identification Test; DUDIT, Drug Use Disorders Identification Test.*

**/** Continuous variables were compared across groups using T-tests, and categorical variables were compared across groups using Chi-squared tests. Mann Whitney U-tests were performed for non-parametric continuous variables.*

From the specified ordinal logistic regression model, the probability of being in each of the outcome groups at one-year follow-up were calculated for specific scores on the independent variable, while holding all other covariates constant. We used the margins command in STATA to find predicted probabilities for the variables included in the ordinal logistic model (Table S3).

Table S3: Estimated probability (Pr)^a^ and 95% confidence interval (95% CI) for chosen values of the independent variables to illustrate the impact of each of the independent variables.

|  | Probability (95% CI) | | |
| --- | --- | --- | --- |
| *Variables* | ECR | P-ECR | No-ECR |
| Gender  Male  Female | 0.25 (0.19 – 0.32)  0.38 (0.29 – 0.46) | 0.23 (0.17 – 0.29)  0.25 (0.19 – 0.31) | 0.52 (0.44 – 0.60)  0.38 (0.29 – 0.46) |
| PAS social childhood  Pas = 0  Pas = 1  Pas = 2  Pas = 3 | 0.35 (0.27 – 0.42)  0.30 (0.24 – 0.36)  0.26 (0.19 – 0.32)  0.22 (0.14 – 0.30) | 0.25 (0.19 – 0.32)  0.25 (0.19 – 0.31)  0.24 (0.18 – 0.30)  0.23 (0.17 – 0.28) | 0.40 (0.32 – 0.48)  0.45 (0.39 – 0.51)  0.50 (0.43 – 0.57)  0.55 (0.45 – 0.66) |
| DUP  0 weeks  30 weeks  60 weeks  200 weeks  500 weeks  1000 weeks | 0.43 (0.31 – 0.54)  0.28 (0.23 – 0.34)  0.26 (0.20 – 0.32)  0.22 (0.15 – 0.29)  0.19 (0.11 – 0.27)  0.17 (0.08 – 0.25) | 0.26 (0.19 – 0.32)  0.25 (0.19 – 0.32)  0.25 (0.19 – 0.31)  0.23 (0.18 – 0.29)  0.22 (0.16 – 0.28)  0.21 (0.15 – 0.27) | 0.32 (0.21 – 0.43)  0.46 (0.40 – 0.53)  0.49 (0.43 – 0.56)  0.55 (0.46 – 0.63)  0.59 (0.48 – 0.69)  0.62 (0.50 – 0.74) |
| Pos. sympt baseline  Pos = 0  Pos = 2  Pos = 4  Pos = 6 | 0.66 (0.50 – 0.83)  0.34 (0.27 – 0.41)  0.11 (0.05 – 0.18)  0.03 (0.00 – 0.06) | 0.20 (0.12 – 0.29)  0.28 (0.21 – 0.35)  0.19 (0.12 – 0.25)  0.07 (0.00 – 0.14) | 0.13 (0.04 – 0.23)  0.38 (0.30 – 0.45)  0.70 (0.58 – 0.81)  0.90 (0.80 – 1.01) |
| Neg. sympt baseline  Neg = 0  Neg = 2  Neg = 4  Neg = 6 | 0.46 (0.31 – 0.60)  0.30 (0.24 – 0.36)  0.17 (0.08 – 0.26)  0.09 (0.00 – 0.19) | 0.25 (0.19 – 0.31)  0.25 (0.19 – 0.31)  0.21 (0.15 – 0.27)  0.15 (0.05 – 0.25) | 0.29 (0.16 – 0.42)  0.45 (0.39 – 0.51)  0.62 (0.49 – 0.75)  0.76 (0.57 – 0.96) |

*Abbreviations: ECR, early clinical recovery; P-ECR, partial early clinical recovery; No-ECR, no early clinical recovery; 95% CI, 95% confidence interval; PAS, premorbid adjustment scale; DUP, duration of untreated psychosis; Pos, positive; neg, negative.*

*^a^Estimated based on ordinal logistic regression (PO) model, 95% confidence interval.*

Table S4: Demographic, premorbid, clinical characteristics based on fulfilling all either CR or TRRIP criteria at 1YFU.

|  | CR by RSWG  at 1YFU  (n=27, 13%) | TR by TRRIP  at 1YFU  (n=35, 17%) | T-test/chi square */Mann Whitney U** analyses | | |
| --- | --- | --- | --- | --- | --- |
|  |  |  | T (x^2*^) | df | P |
| Gender (male), n (%) | 12 (44.4) | 26 (74.3) | 5.72* | 1 | **.021** |
| Age, years: mean (SD) | 26.7 (6.9) | 25.7 (5.7) | 0.619 | 60 | .538 |
| PAS social childhood | 0.48 (0.8) | 1.38 (1.6) | -2.73 | 59 | **.008** |
| PAS academic childhood | 1.07 (0.9) | 1.71 (1.3) | -2.13 | 59 | **.038** |
| Diagnosis (narrow schizophrenia), n (%) | 9 (33.3) | 29 (82.9) | 15.76* | 1 | **<.001** |
| DUP, median (range) | 9.0 (532) | 40.0 (415) | 680.0** | 1 | **<.001** |
| GFS baseline | 56.8 (13.0) | 37.0 (6.4) | 7.9 | 60 | **<.001** |
| PANSS pos baseline | 1.56 (0.7) | 2.80 (0.9) | -6.1 | 60 | **<.001** |
| PANSS neg baseline | 1.80 (0.8) | 2.39 (1.0) | -2.5 | 60 | **.007** |
| PANSS dis baseline | 1.40 (0.5) | 2.16 (0.8) | -4.7 | 60 | **<.001** |
| PANSS depr baseline | 2.31 (0.9) | 3.30 (0.9) | -4.2 | 60 | **<.001** |
| PANSS excit baseline | 1.19 (0.3) | 1.43 (0.6) | -2.0 | 60 | **.018** |
| Audit score, median (range) | 5.0 (15) | 3.0 (22) | 346.5** | 1 | .101 |
| Dudit score, median (range) | 0.0 (18) | 0.0 (28) | 472.0** | 1 | .776 |

*Abbreviations: CR, clinical recovery; RSWG, early remission in schizophrenia working group; TR, treatment resistant; TRRIP, treatment response and resistance in schizophrenia; 1YFU, one-year follow-up; PAS, premorbid adjustment scale; DUP, duration of untreated psychosis; GFS, Global Functioning Scale; PANSS, Positive and Negative Syndrome Scale; AUDIT, Alcohol Use Disorder Identification Test; DUDIT, Drug Use Disorders Identification Test.*

**/** Continuous variables were compared across groups using T-tests, categorical variables were compared across groups using Chi-squared tests. Mann Whitney U-tests were performed for non-parametric continuous variables.*

We included the demographic, premorbid and illness onset clinical characteristics associated at p <.05 with outcome groups in the initial analyses in a binary logistic regression analysis (Table S5, n=59).

Table S5: Logistic regression analysis fulfilling either CR or TRRIP criteria.

| Parameter | OR | 95% CI | | Wald | Sig. |
| --- | --- | --- | --- | --- | --- |
| Gender | 5.80 | .88 | 38.0 | 3.36 | .067 |
| Pas social childhood | 1.27 | .67 | 2.39 | .54 | .461 |
| DUP (log transformed) | 1.72 | 1.03 | 2.87 | 4.24 | **.040*** |
| Pos symptoms baseline | 8.33 | .58 | 3.80 | 10.60 | **.001*** |
| Neg symptoms baseline | 1.48 | .58 | 3.80 | .68 | .411 |

*Abbreviations: CR, clinical recovery; TRRIP, treatment response and resistance in schizophrenia; OR, odds ratio; 95% CI, 95% confidence interval; PAS, premorbid adjustment scale; DUP, duration of untreated psychosis; Pos, positive; neg, negative. Cox and Snell R2 = .506, Nagelkerke R2 = .680.*

Table S6: Information about first choice of antipsychotic treatment

| **First antipsychotic medication used** | **ECR**  **(n=61)** | **P-ECR**  **(n=49)** | **No-ECR**  **(n=97)** | **Total**  **(n=207)** |
| --- | --- | --- | --- | --- |
| Olanzapine n (%) | 31 (50.8) | 20 (40.8) | 43 (44.3) | 94 (45.4) |
| Aripiprazole n (%) | 10 (16.4) | 10 (20.4) | 16 (16.5) | 36 (17.4) |
| Risperiodone n (%) | 3 (4.9) | 4 (8.2) | 8 (8.2) | 15 (7.2) |
| Quetiapine n (%) | 5 (8.2) | 9 (18.4) | 9 (9.3) | 23 (11.1) |
| Ziprasidon n (%) | 4 (6.6) | 2 (4.1) | 3 (3.1) | 9 (4.3) |
| Amisulpride n (%) | 0 (0.0) | 0 (0.0) | 2 (2.1) | 2 (1.0) |
| Perfenazin n (%) | 1 (1.6) | 0 (0.0) | 3 (3.1) | 4 (1.9) |
| Paliperidone n (%) | 1 (1.6) | 0 (0.0) | 1 (1.0) | 2 (1.0) |
| Not used n (%) | 6 (9.8) | 4 (8.2) | 12 (12.4) | 22 (10.6) |

*Abbreviations: ECR, early clinical recovery; P-ECR, partial early clinical recovery; No-ECR, no early clinical recovery.*

Table S7: Information about second choice of antipsychotic treatment

| **Second antipsychotic medication used** | **ECR**  **(n=61)** | **P-ECR**  **(n=49)** | **No-ECR**  **(n=97)** | **Total**  **(207)** |
| --- | --- | --- | --- | --- |
| Olanzapine n (%) | 0 (0.0) | 3 (6.1) | 7 (7.2) | 10 (4.8) |
| Aripiprazole n (%) | 17 (27.9) | 7 (14.3) | 20 (20.6) | 44 (21.3) |
| Risperiodone n (%) | 2 (3.3) | 5 (10.2) | 6 (6.2) | 13 (6.3) |
| Quetiapine n (%) | 9 (14.8) | 1 (2.0) | 8 (8.2) | 18 (8.7) |
| Ziprasidon n (%) | 1 (1.6) | 1 (2.0) | 3 (3.1) | 5 (2.4) |
| Amisulpride n (%) | 0 (0.0) | 1 (2.0) | 1 (1.0) | 2 (1.0) |
| Perfenazin n (%) | 0 (0.0) | 2 (4.1) | 1 (1.0) | 3 (1.4) |
| Paliperidone n (%) | 0 (0.0) | 1 (2.0) | 3 (3.1) | 4 (1.9) |
| Not used n (%) | 6 (9.8) | 4 (8.2) | 12 (12.4) | 22 (10.6) |

*Abbreviations: ECR, early clinical recovery; P-ECR, partial early clinical recovery; No-ECR, no early clinical recovery.*
